# Epidemiology of HPV in HIV-Positive and HIV-Negative Fertile Women in Cameroon, West Africa

**DOI:** 10.1155/2009/810596

**Published:** 2010-02-09

**Authors:** Andrew J. Desruisseau, Delf Schmidt-Grimminger, Edith Welty

**Affiliations:** ^1^Division of Infectious Diseases, Department of Internal Medicine, Meharry Medical College, 1005 Dr. DB Todd Jr Boulevard, Nashville, TN 37208, USA; ^2^Department of Obstetrics and Gynecology, Sanford School of Medicine, The University of South Dakota, Vermillion, Sioux Falls, SD 57105, USA; ^3^Clinical Research Division, Avera Research Institute, Sioux Falls, SD 57105, USA; ^4^Cameroon Baptist Convention Health Board, Bamenda, Cameroon

## Abstract

*Background*. HPV types vary by country and HIV status. There are no data on the prevalent HPV genotypes from Cameroon. *Methods*. We conducted a cross-sectional, observational study on 65 Cameroonian women. Samples were sent for HPV genotyping and Thin Prep analyses. *Results*. 41 out of 61 samples tested (67.2%) had HPV subtypes detected. The most common high risk types encountered were: 45 (24.6%) and 58 (21.5%). HIV-positive women were more likely to test positive for any HPV (*P* = .014), have more than one HPV subtype (*P* = .003), and to test positive for the high risk subtypes (*P* = .007). Of those with high risk HPV, HIV-positive women were more likely to have Thin Prep abnormalities than HIV-negative women (*P* = .013). *Conclusions*. Oncogenic HPV subtypes 45 and 58 were more prevalent than those subtypes carried in the quadrivalent vaccine. Further studies are needed to assess whether the current vaccine will be effective in this region.

## 1. Introduction

Cervical cancer is the second most common cancer in women worldwide, but it is the first in most developing countries, where 80% of cases occur [1] and only 5% of the at-risk population are screened [2, 3]. Further, cervical cancer is the leading cancer-related cause of death in African women [4]. Certain human papillomavirus (HPV) genotypes, such as HPV 16 and 18, have clearly been shown to be obligate causative agents in the development of cervical cancer [5]. Only women with persistent infection with oncogenic subtypes develop cervical cancer.

There are at least 15 high-risk (oncogenic) subtypes of HPV [6]. Overall, HPV-16 and HPV-18 account for approximately 70% of all cervical cancer diagnosed worldwide each year [5, 7]. HPV genotypes 6 and 11 are associated with genital warts (condyloma aciminata) and are not linked to cervical cancer or its precursor lesions. Several HPV vaccines, including the now commercially available vaccine against HPV genotypes 6, 11, 16, and 18, have been shown to reduce the acquisition of infection and clinical disease caused by these HPV genotypes and likely will lead to reductions in cervical cancer and its precursor lesions [8–10].

HPV genotypes, however, differ greatly in their geographic distribution. In sub-Saharan Africa, for instance, where two thirds of the world's HIV-infected population live, HPV types vary by country and HIV status and differ significantly from HPV types seen in other regions of the world, possibly in part due to geographic and genetic variation in HLA haplotypes [11–15]. Multiple prospective studies have demonstrated that women with HIV infection are much more likely than HIV-negative women to have persistent HPV, often with oncogenic subtypes [16–18]. It has been estimated that a vaccine against HPV types 16 and 18 would theoretically prevent 71% of cervical cancers worldwide, but would preferentially favor Asia and North America over Africa and South America [14]. To our knowledge, there are no data in Cameroon on HPV prevalence or genotypes. 

This pilot study seeks to describe the prevalence of HPV genotypes in HIV-positive and HIV-negative fertile Cameroonian women, to determine risk factors for HPV utilizing a culturally appropriate questionnaire and to assess the association of HPV types, squamous intraepithelial lesions (SIL), HIV, and other reproductive tract infections.

## 2. Materials and Methods

### 2.1. Study Population and Setting

This study was approved by both the institutional review board of Meharry Medical College and the Cameroon Baptist Convention Health Board (CBCHB). Between June 2005 and March 2006 sixty-five women age eighteen and over were enrolled from two CBCHB clinical sites Baptist Hospital Mutengene and Etoug-Ebe clinic in Yaounde. Women were recruited from both antenatal clinics and existing HIV positive women's support groups. Women were excluded if they had never been pregnant, had a hysterectomy, were in active labor, had abnormal bleeding, had placenta previa, or were unwilling to undergo HIV testing. The study was explained to patients either in English, French, or Pidgin English. Similarly, informed consent was obtained in English or French by clinic nurses or midwives.

Participants were then asked to complete a questionnaire designed to elicit demographic characteristics, obstetric, contraceptive and sexual history, and current genital tract symptoms. General, pelvic, and speculum examination and sample collection were performed. Only support group mothers also underwent bimanual exams. Women, and their partners if necessary, were treated free of charge for reproductive tract infections (RTIs) when clinically indicated in accordance with World Health Organization published guidelines [19]. Participants were counseled on low-risk sexual behavior and provided with contraceptive counseling, and/or condoms if needed. Return appointments and referrals were made as appropriate.

### 2.2. Samples Taken and Laboratory Methods

#### 2.2.1. Cervical Mucus Samples

An ophthalmic sponge, Zyomed Treace (Orlando, FL), was placed directly into the cervical os. The spears were allowed to absorb cervical secretions for approximately 30 seconds after which time they were placed in an eppendorf tube and immediately placed on ice, before they were transported on dry ice to the University of South Dakota for possible later cytokine analysis under the direction of Dr. Delf Schmidt-Grimminger.

#### 2.2.2. Cervical Cytology

After introduction of a clean, nonlubricated speculum, the endocervical secretions were collected as previously described in [20] with an Ayres spatula and cervical brush (or Dacron swab, if the patient was in the last trimester of pregnancy) and placed it in PreservCyt (Cytyc Corporation, Marlborough, MA). Thin Prep smears were sent to the University of Kansas Medical Center, where they were reviewed by a cytotechnologist under the direction of Dr. Patricia Thomas, M.D., a board certified pathologist. Thin prep abnormalities were classified according to the Bethesda classification system [21]. Testing for Neisseria gonorrhoeae (NG) and Chlamydia trachomatis (CT) was performed off of the Thin Prep sample as previously described [22].

#### 2.2.3. HPV PCR

Next, a sample for HPV testing was collected with a cervical brush or Dacron swab, placed in a Specimen Treatment Medium (STM; Digene Corporation, Silver Spring, MD), and sent to the South Dakota Health Research Foundation at the University of South Dakota, where the HPV typing proceeded under the direction of Dr. Delf Schmidt-Grimminger. An aliquot of the cervical sample was processed as described previously [23]. Briefly, the sample was precipitated and centrifuged, and the pellet dried and resuspended. HPV detection and typing was carried out using a polymerase chain reaction- (PCR-) based reverse-line strip test for HPV types 6, 11, 16, 18, 26, 31, 33, 35, 39, 40, 42, 45, 51–59, 66, 68, 73, 82, 83, and 84. B-globin was used as an internal control as previously described in [24]. If the participants consented, any residual specimens were stored for possible additional testing related to reproductive tract infections.

All transported specimens were frozen at 0 degrees Celsius due to logistical problems and shipped at −20 C using dry ice according to international standards.

#### 2.2.4. Additional Testing

Only if a patient complained of, or had signs of an abnormal vaginal discharge or pelvic infection, did we collect a vaginal pool specimen and place it in a test tube containing saline and send it to the on site lab to test for pH, wet saline preparation for *T*. v*aginalis *and bacterial vaginosis, and potassium hydroxide (KOH) prep for Candida. Bacterial vaginosis was defined as the presence of “clue cells” (epithelial cells completely covered with coccobacilli to the extent that the cell borders are obscured) constituting more than 20% of vaginal epithelial cells per high power field, and a pH of >4.5. All identified infections were treated free of charge according to WHO guidelines [19].

#### 2.2.5. Serology

All participants without documentation of HIV or syphilis tests within 6 months underwent testing after pretest counseling. A serial testing protocol of up to 3 rapid HIV tests was used to determine HIV status as previously described in [25]. Our lab used the Determine HIV-1/2 (Abbott Laboratories, Tokyo, Japan) and the Determine Syphilis test, both of which produce results in 15 minutes. Patients with a negative Determine HIV result were counseled as negative, were told about the “window period,” and advised to have a repeat HIV test in 3–6 months. The laboratory performed a SD BIOLINE HIV 1/2 3.0 rapid HIV test (Standard Diagnostics, Inc., Kyonggi-do, Korea) on all patients with a positive Determine test. If Determine and Bioline tests were both positive, the patient was posttest counseled as positive. If Determine and Bioline test results were discrepant, the laboratory performed a 3rd “tie breaker” test (Hexagon HIV, Human GmbH, Germany) and the patient was counseled according to the result of that test.

### 2.3. Data Analysis

Data were analyzed using SPSS 15.0 (SPSS Inc, Chicago, IL, USA). Simple frequency analyses, Fisher's exact test, and Chi-square tests were used. *P*-values <.05 were considered significant.

## 3. Results

Sixty-five women were enrolled in the study, 36 in the antenatal group and 29 in the support group. The median age was 27 years in the antenatal group and 29 in the support group, respectively. 8.3% of the antenatal participants tested positive for HIV while 100% of the support group participants tested HIV positive. The women did not differ with respect to education, marriage status, husband's number of wives, age at sexual debut, or cell phone ownership which was used as a surrogate marker for income. However, over 50% of the antenatal participants reported never using condoms and over 80% reported never or only seldom using condoms during sex. Similarly, only 35% of known HIV positive women participants from the support group reported using condoms often (Table 1).

**Figure 1:**
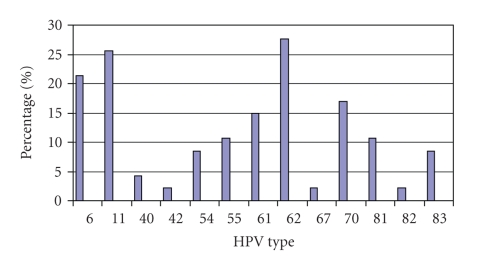
Low risk HPV type distribution.

**Figure 2:**
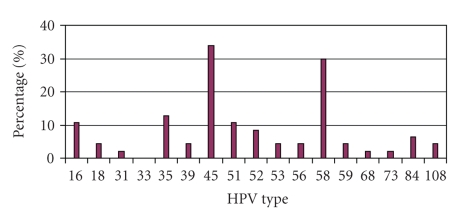
High risk HPV type distribution.

Overall, out of the 65 women who signed consent forms, 4 had no evidence of specimens in their transport media. 41 of the 61 samples tested (67.2%) had HPV subtypes detected. The most common low risk types detected out of all analyzed specimens were: 62 (20.0%), 11 (18.5%), 6 (15.4%), 70 (12.3%), and 61 (10.8%). The most common high risk types encountered were: 45 (24.6%) and 58 (21.5%). The data is presented by frequency of HPV type detected as a percentage of all women in the study (HPV positive and negative) as shown in Figures 1 and 2.

Thirty-five out of the 41 women (85.4%) who tested positive for HPV had more than one HPV subtype identified, while 32 out of 41 (78%) had at least one high risk subtype identified. Twenty-eight out of 41, or 68.3%, with HPV isolated had subtype 6, 11, 16, or 18 which are currently carried in the commercially available HPV vaccine. Those who tested positive for the high risk subtypes carried in the current vaccine (subtypes 16 or 18) totaled 7 out of 41, or 17.1% (see Table 2).

In comparing HIV-negative to HIV-positive participants, HIV positive women were more likely to have tested positive for any HPV, have more than one HPV subtype, and to test positive for the high risk HPV subtypes than their HIV-negative counterparts when analyzed using the Chi-Square statistic with a significant *P*-value defined as <.05. They were also more likely to have one of the HPV subtypes covered in the current quadrivalent HPV vaccine, although not when including subtypes 16 and 18 only.

Atypical squamous cells of unknown significance (ASCUS), low grade squamous intraepithelial lesions (LGSILs), and high grade intraepithelial lesions (HGILs) represent increasing degrees of HPV-related cellular change and are precancerous lesions. Women who were HIV positive with high risk HPV were more likely than HIV negative women with high-risk HPV to have abnormalities on thin prep evaluation, even after controlling for the effect of high risk HPV on thin prep abnormalities using Chi-Square analysis (*P*-value for HIV positive .013, for HIV negative .235) (Table 3). Surprisingly, we did not encounter any high-grade intraepithelial lesions in either group.

## 4. Discussion

Our pilot study results add to a rich body of literature demonstrating that HPV types other than those included in the quadrivalent vaccine are being encountered in many African countries. In a recent international meta-analysis, including 5 African studies, Clifford et al. found that the proportion of HPV prevalence attributable to HPV-16 was lower in HIV-positive women, including those with HGSIL, than in the general population and that other high-risk types (e.g., HPV-18, 31, 33, 51, 52, and 58) were also prevalent [26, 27]. Muñoz et al. demonstrated from pooled analyses of data from an international survey of HPV types in cervical cancer and from a multicenter case-control study that a higher proportion than usual of HPV-45 was found in sub-Saharan Africa [14]. Similarly, Sahasrabuddhe et al performed HPV typing on 145 nonpregnant HIV-positive Zambian women and found that, among the high-risk types, HPV types 52, 58, and 53 were more common than HPV types 16 and 18 [28]. As many others have recently demonstrated, HPV subtypes other than 16 and 18 are clearly playing a role in international HPV prevalence [29–32].

We found that the known oncogenic subtypes 45 and 58 were significantly more prevalent than subtypes 16 and 18. With increasing numbers of HIV-infected women in sub-Saharan Africa now accessing life-prolonging antiretroviral therapy, these women may now live long enough to be at higher risk for developing cervical cancer [33, 34]. Our finding has implications in the incipient application of the prophylactic HPV vaccine containing the high risk subtypes 16 and 18 in countries where cervical cancer prevalence continues to be much higher than it is here in the United States [35]. There has been encouraging yet far from conclusive data to suggest that, because of closely related DNA genomes between HPV types, there may be HPV type cross-protection afforded by the quadrivalent vaccine [36, 37].

We noted a prevalence of HPV in our study of 67.2% with 85.4% having more than one HPV type and 78.0% having one of the high risk subtypes. This is at the higher end of previous comprehensive estimates in similar HIV-positive female populations [29–32, 38, 39] which have reported 12–79% of study participants with more than one HPV subtype. Our high prevalence may be reflective of the immunosuppression caused by concomitant infection with HIV. Unfortunately, our study did not include measurements of either CD4 counts or HIV viral RNA to further investigate this possibility. Most HIV-positive women in our study never had such testing and thus analyses between HPV and HIV progression were not feasible.

Although our study set out to determine possible interactions between HPV, HIV, and other reproductive tract infections, we were not able to accurately assess our participants for gonorrhea or chlamydia due to a delay in transferring the Thin Prep samples to appropriate testing media within 7 days, as is recommended by the manufacturer (Cobas Amplicor CT/NG Test, Hoffmann-La Roche Inc, Basel, Switzerland). However, we did not detect a significant number of syphilis, trichomonas, bacterial vaginosis, or vaginal candidiasis infections (data not shown). Our study is further limited by the small number of participants enrolled as it was designed as a pilot study.

We were still able to demonstrate, however, that HIV-positive women are more likely to test positive for HPV, have more than one HPV type, and to be infected with high risk HPV than their HIV-negative counterparts. This may be explained by the impact of HIV on the host's immune system resulting in impaired HPV clearance, leading to chronic HPV infection. The higher number of HPV types encountered by the HIV-positive women in our study may be attributed to HIV-mediated upregulation and persistence of HPV [39–41] or repeated HPV exposure in the setting of severe immunosuppresion.

Even after controlling for the effect of high risk HPV on Thin Prep findings, HIV was still associated with ASCUS or LGSIL on Thin Prep analyses. We cannot explain why no participants demonstrated HGSIL, as previous studies from Senegal (11%) and Burkina Faso (3.8%) did demonstrate a significant number of HIV positive women with HR-HPV to have HGSIL [30, 38]. Fortunately, ASCUS or LGSIL lesions are more likely to regress rather than progress towards cervical cancer. Our study was not designed to conduct repeat testing for HPV which would allow us to further assess progression of cervical lesions or assess whether previously noted HPV types persisted or were cleared and whether new HPV types would be encountered.

Overall our pilot study includes Cameroon in the growing list of African countries where HPV types differ from those currently included in the prophylactic vaccine. With increasing numbers of women living with HIV longer, cervical cancer has the potential to increase in incidence and prevalence in countries such as Cameroon. Further studies are needed to assess whether the current vaccine will be effective in cervical cancer prevention programs in this region.

## Figures and Tables

**Table 1:** Demographics.

	Antenatal group *N* = 36	Support group *N* = 29	*P*-value
Age, median (range)	27 (23–36)	29 (26–32)	.960
HIV positive %	8.3	100.0	.001
Cell phone %	75	58.6	.550
School in median no. Yrs, (range)	12 (0–21)	11 (6–19)	.164
Married %	80.6	75.9	.522
No. wives, median (range)	1.0 (1–3)	1.0 (1–2)	.524
Age at sexual debut, median (range)	18 (13–37)	18 (13–21)	.310
No. partners, median (range)	1.0 (0–7)	1.0 (0–3)	.325
Condoms,%			.001
Never	51.5	10.7	
Seldom	30.3	35.7	
Often	12.1	35.7	
Don't Know	6.1	17.9	

**Table 2:** Influence of HIV on HPV.

	HIV NEG	HIV POS	*P*-value
Any HPV (%)	15/29 (51.7)	26/32 (81.3)	.014
>1 HPV TYPE (%)	11/29 (37.9)	24/32 (75.0)	.003
High-risk HPV (%)	10/29 (34.5)	22/32 (68.8)	.007
Types 6, 11, 16, or 18 present (%)	8/29 (27.6)	20/32 (62.5)	.006
Types 16 or 18 present (%)	1/29 (3.5%)	6/32 (18.8)	.061

**Table 3:** Thin prep findings.

	NEG	ASCUS	LGSIL	HGSIL
HIV POS				
HR HPV+	10	6	6	0
HR HPV−	10	0	0	0
HIV NEG				
HR HPV+	5	2	2	0
HR HPV−	16	1	2	0

HIV+* P = *.013, HIV− *P = *.235, ASCUS: atypical squamous cells of unknown significance, LGSIL: low-grade squamous intraepithelial lesion, HGSIL: high-grade squamous intraepithelial lesion, HR: high risk.
